# Targeting Innate Immunity in Breast Cancer Therapy: A Narrative Review

**DOI:** 10.3389/fimmu.2021.771201

**Published:** 2021-11-25

**Authors:** Yanqi Ye, Chun Xu, Fengqian Chen, Qi Liu, Ning Cheng

**Affiliations:** ^1^ Zenomics. Inc. Magnify at California NanoSystems Institute, Los Angeles, CA, United States; ^2^ School of Dentistry, The University of Queensland, Brisbane, QLD, Australia; ^3^ School of Medicine, University of Maryland, Baltimore, MD, United States; ^4^ School of Medicine, Johns Hopkins University, Baltimore, MD, United States; ^5^ Department of Otolaryngology - Head and Neck Surgery, University of California at San Francisco, San Francisco, CA, United States

**Keywords:** innate immunity, breast cancer, cancer immunotherapy, clinical studies, vaccine adjuvants

## Abstract

Although breast cancer has been previously considered “cold” tumors, numerous studies are currently conducted to explore the great potentials of immunotherapies in improving breast cancer patient outcomes. In addition to the focus on stimulating adaptive immunity for antitumor responses, growing evidence showed the importance of triggering host innate immunity to eradicate established tumors and/or control tumor metastasis of breast cancer. In this review, we first briefly introduce the breast tumor immune microenvironment. We also discuss innate immune targets and pathways and mechanisms of their synergy with the adaptive antitumor response and other treatment strategies. Lastly, we review clinical trials targeting innate immune pathways for breast cancer therapies.

## 1 Introduction

Breast cancer is considered one of the leading causes of death in women. The American Cancer Society (ACS) predicted that nearly 280,000 new breast cancer cases would be diagnosed in 2020. Over 40,000 breast cancer-related deaths can be present in the same year (1). Breast cancer disease is well known for its phenotypical heterogeneity. It is usually classified into three subtypes that display unique cellular and molecular patterns: luminal ER-positive, HER2-like, and basal-like subtype (2, 3). In general, breast tumors mainly consist of malignant cells, extracellular matrix (ECM), and stromal components, including infiltrating immune cells that shape tumor initiation. The malignant cells undergo a constant evolution during tumor development to form a specialized tissue architecture with a dissociated ECM and cancer-associated inflammation (4).

## 2 The Importance of Innate Immunity in Breast Cancer

### 2.1 Overview of Immunology in Breast Cancer

Notably, cancer-associated inflammation, including cancer-intrinsic inflammation and cancer extrinsic inflammation, is present at different stages of breast tumorigenesis (5–8). Cancer-intrinsic inflammation is usually triggered by cancer-initiating mutations that are genetically stable and can be predictable for drug treatment response or resistance (7). Leukocytes are recruited and migrated to the cancer site, further undergoing activation during tumor progression. However, cancer-extrinsic inflammation is associated with various exogenous risk factors, such as bacterial or viral infections, obesity, excessive alcohol consumption, tobacco smoking, hormone therapies, autoimmune diseases, overexposure to radiation, *etc.* (8). Both cancer-intrinsic and cancer-extrinsic inflammation contribute reciprocally to immunosuppression, which provides a favorable environment for malignant progression.

### 2.2 Breast Cancer Immune Microenvironment

**Figure 1:**
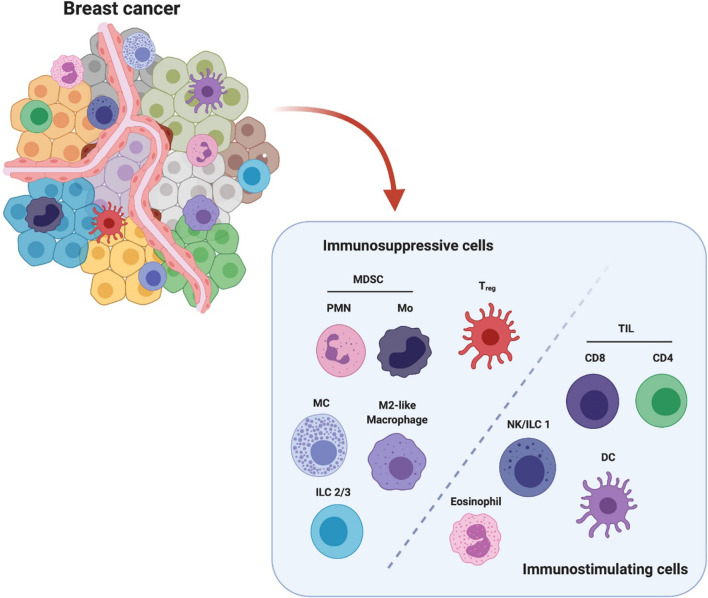
Breast cancer microenvironment is populated by diverse infiltrating immune cells. These immune cells are categorized into immunosuppressive population and immunostimulating population (e.g.) according to their major characteristics in modulating breast cancer. The immunosuppressive cells include polymorphonuclear myeloid-derived suppressor cells (PMN-MDSCs), monocytic MDSC (Mo-MDSC), regulatory T cells (Treg), mast cells (MC), M2-like macrophages and type 2/3 innate lymphoid cells (ILC 2/3). The immunostimulating cells include tumor infiltrating lymphocytes CD8+ and CD4+ T cells, nature killer cells/type 1 innate lymphoid cells (NK/ILC1), dendritic cells (DCs) and eosinophils. This figure is reproduced with permission from “The Crosstalk Between Tumor Cells and the Immune Microenvironment in Breast Cancer: Implications for Immunotherapy, doi.org/10.3389/fonc.2021.610303” Copyright ©2021 Frontiers.

The microenvironment of breast cancer is highly inflammatory. It consists of a broad spectrum of stromal components, including mesenchymal-derived fibroblasts, pericytes, vascular structure, infiltrating immune cells, cytokines and growth factors, which coordinately enable carcinogenesis and breast tumor progression (9–12). The breast tumor stroma complex also supports neovascularization, as well as functionally modulating the immune cells, cytokines and inflammatory cascade. These together orchestrate the formation of solid breast tumors (5, 13, 14). Breast tumor microenvironment is populated by diverse innate immune cells (macrophages, dendritic cells, natural killer cells, myeloid-derived suppressor cells, mast cells, and granulocytes) and adaptive immune cells (T and B lymphocytes, NKT cells) (**Figure 1**). These infiltrating immune cells are either originated from residential mammary tissue associated populations or recruited from the periphery or draining lymphoid organs. The types of inflammatory cells and their roles have been well identified in both animal and human breast cancer studies (6, 10, 15, 16). For example, human monocytes/macrophages have been demonstrated to produce EGF receptor (EGFR) ligands and stimulate STAT3 signaling pathway to promote mammary tumor cell invasion, which in turn facilitated TAMs infiltration depending on the secreted CSF1 and CXCL12 (17, 18). Another study has reported that inflammatory breast cancer patients express a high level of IL8 and growth-regulated oncogene (GRO) chemokines that activate STAT3, which induce the formation of immune-suppressive M2-like macrophages and cancer mesenchymal cells (15). In murine 4T1 metastatic breast tumors, tumor entrained neutrophils have been shown to mediate anti-metastasis in the lung by inducing oxidative stress and CCL2 (19). Furthermore, a cohort study of approximately 3000 patients suggested that circulating leukocyte profiles may serve as biomarkers to predict breast cancer risk (16). Collectively, these innate immune cells can regulate tumor cell invasion, differentiation, and disease development.

Moreover, recent findings suggested that infiltrating immune cells are associated with treatment response, which can potentially predict clinical outcomes in breast cancer patients. For instance, tumor-infiltrating CD8^+^ cytotoxic lymphocytes were positively correlated with tumor stage and improved survival rate (20). In contrast, the infiltrating regulatory T cells correlated with poor treatment outcomes in both ER-negative and positive breast cancers (21, 22). In particular, infiltrating innate immune cells build a cellular network and rigorously regulates the antitumor response *via* direct tumor killing and/or bridging and activating the adaptive antitumor immunity (12, 13). Here, we focus on discussing the innate immune cells and therapeutic targets and pathways for developing translational antitumor treatment.

### 2.3 Impact of Innate Immunity in Breast Cancer

Innate immune populations play key roles during cancer evolution, specifically contributing to early responses against cancer-associated inflammation. They also can initiate a complete and vigorous antitumor response *via* regulating critical pathways for enhanced priming of adaptive immunity. In breast cancer, malignant cells primarily encounter different innate immune cells to trigger specific downstream cascades. Classic activation of adoptive T cell response needs three signals transmitted from innate antigen-presenting cells (APCs) (23). For instance, professional APCs, including DCs and macrophages, first recognize and bind to the dying breast tumor cells or release tumor-associated antigens through pattern recognition receptors (PRRs) (24, 25). PRRs can identify and recognize the damage-associated molecular patterns (DAMPs), which are derived from the tumor or dying cells to drive cancer intrinsic inflammation. They can also detect foreign pathogen-associated molecular patterns (PAMPs), including the microbes encountered during extrinsic cancer inflammation (24, 26). Upon binding of DAMPs or PAMPs, APCs internalize and load tumor antigen or DNA into upregulated major histocompatibility complex (MHC). They form MHC-peptide complexes, binding to the T cell receptor (TCR) as the first activation signal (27). To further activate adaptive lymphocytes for tumor antigen-specific cytotoxic killing, APCs are required to display co-stimulatory molecules of both CD80 and CD86 and recognize the CD28 expressed on lymphocytes as the second signal (28). The secreted cytokines (*e.g.*, TGF-β, IL-1β, IL-6, IL-23, IL-12, and interferon) act as the third signal to support repolarization of the activated lymphocytes into specific subsets, which can induce distinct proinflammatory (antitumoral) or anti-inflammatory (pro-tumoral) responses (29).

Breast cancer, including ER-positive and ER-negative subsets, has heterogeneous patterns of innate immune infiltration. In ER-positive breast tumors, NK cells and neutrophils have been found as major innate immune populations (9, 30). Tumor cells are known to escape the host immune attack by self down-regulating MHC Class I antigens, which can be recognized by NK cells and potentially induce antitumor effect *via* the release of stimulatory cytokines and chemokines (31). Interestingly, the cooperative interplay between NK cells and intratumoral type I conventional dendritic cells (cDC1) can affect tumor microenvironment in response to checkpoint immunotherapy (32, 33). Others have reported the use of eosinophils, monocytes, and mast cells as prognostic biomarkers in breast cancer, while tumor-associated macrophages (TAMs) have been associated with poor prognosis (34–37). In ER-negative breast tumors, a large proportion of TAMs and mature mast cells was correlated with an increased risk of metastasis and poor prognosis. Meanwhile, cDC1s responses are significant against breast cancer. Despite their small proportion within breast tumors, cDCs are associated with favorable immune infiltrate and better prognosis (36, 38–40). Recently studies have shown that cDC1 induce antitumor responses by facilitating Th1 immunogenic microenvironment, which correlates with the levels of IL-12, IFN-γ, and cytotoxic lymphocyte–recruiting chemokines (41–45). These findings suggest the impact of innate immunity in modulating the immunosuppressive microenvironment that can improve breast cancer immunotherapeutic outcomes. Therefore, we believed that explore the defining characteristics of the innate immunity provided a much-needed bridge for translational breast cancer research.

## 3 Preclinical Studies Targeting Innate Immunity for Breast Cancer Therapy

Innate immunity is the primary defense line of the host against mammary tumor progression. Upon encountering dying breast tumor cells released components, immune surveillance is established for direct antitumor activities, including immune recognition and suppression (46, 47). Specifically, the anticancer innate immune cells participate in releasing cytotoxic contents to directly kill target breast tumor cells, engaging professional antigen-presenting cells to detect and remove breast tumor cells, and further activating the phagocytosis process or alternative complement pathways. Thus, modulating innate immunity paves the way for developing innovative cancer therapeutic interventions in preclinical settings of breast carcinoma.

### 3.1 Colony-Stimulating Factors

Colony-stimulating factors (CSF) are hematopoietic cytokines that regulate the proliferation, maturation, and apoptosis of myeloid cells (48). Based on their origins, CSF can be classified as macrophage CSF (M-CSF), granulocyte CSF (G-CSF), granulocyte-macrophage CSF (GM-CSF), and interleukin-3 (IL-3). In breast cancer, CSFs have been reported to play different roles in various stages of tumor immunity. One recently study has shown that CSFs derived from mammary tumor cells primarily regulate myeloid cell arginase 1 expression and form the immunosuppressive microenvironment while inhibiting host anti-tumor immunity (49). In contrast, others have found that CSFs involved in triggering the antigen presentation in the tumor microenvironment to harness innate immunity for antitumor responses. For example, GM-CSF can recruit DCs to the tumor site and undergo cell proliferation and maturation there, and matured DCs are optimally equipped with antitumor immunity (50). They further infiltrate the tumors to stimulate antigen presentation and activate adaptive immunity (30). GM-CSF has been reported to increase the percentage of DCs in tumor-draining lymph nodes and enhance tumor-specific immunity for treatment (51). GM-CSF secreting tumor cells were modulated as a vaccination approach to completely protect the mice after challenging with live parental breast cancer cells (52).

Furthermore, the abundance of M-CSF1 and its receptor (CSF-1R) expressed on TAMs has been correlated to poor clinical outcomes in breast cancer (53). Consistent with this finding, CSF-1R blockade by using small molecular inhibitors and monoclonal antibodies has been applied to deplete TAMs in breast cancer (54). However, Hollmen et al. discovered that anti-CSF-1R treatment could facilitate 4T1 breast tumor cell metastasis to lung and lymph nodes, since 4T1 tumors express high levels of G-CSF (55). Therefore, understanding the sophisticated mechanism of CSF/CSF-1R interaction between tumor and innate immunity can guide to select CSF targeting agents for clinical treatment.

### 3.2 Interferons

Interferons (IFNs), including type I and II IFNs, are involved in regulating immune response in breast cancer. Activation of the type I IFNs (IFN-α/β) signaling pathway can cause cancer cell apoptosis and cellular senescence, and breast tumor cell secreted type I IFNs has been shown to induce anti-metastatic response. However, type I IFNs also attributed to drug resistance specifically in triple negative breast cancers (TNBC) and inflammatory breast cancer (IBC) (56, 57). Type II IFNs (IFN-γ), on the other hand, has been reported to cause tissue damage in response to tumor hyperproliferation. The initiation of tissue damage signals, such as IFN-γ, triggers the innate immune response by recruiting NK cells and T helper cells for differentiation (58). Previous studies have reported the essential roles of IFN-γ signals in polarizing TAMs phenotypes (59) and modulating the MHC I expression level on breast tumor cells (60). For instance, in an epidermal growth factor receptor (HER)2-positive breast tumor model, IFN-γR1^low^ expressing tumor cells successfully escaped from immune surveillance and stayed dormant from tumor elimination (61).

IFN-based therapy has shown promising outcomes in multiple murine breast cancer models (4T1 and 66cl4), specifically by regulating the metastatic spread of tumor cells to the bone (62, 63). The administration of type I IFN *via* intravenous injection could reduce tumor burden, stimulate NK cells and effector CD8^+^ T cells while dampening the immunosuppressive MDSCs in the blood and bone marrow. Notably, these results could limit tumor outgrowth in remote site and eventually increase the metastasis-free survival rate. Clinical trials using IFNs in the solid tumor showed some efficacies. However, the induced moderate-to-severe toxicity issues could not be neglected (64, 65).

### 3.3 Vaccine Adjuvants

Adjuvants are derived from DAMPs and/or PAMPs to improve the potency of breast cancer vaccines and treatment by inducing robust innate immune responses. They can stimulate innate immune cells, such as DCs, by binding to the expressed PRRs, including toll-like receptors, C-type lectin receptors, and retinoic acid-inducible gene I-like receptors, nucleotide-binding oligomerization domain-like receptors, or cytosolic STING-dependent DNA sensors (66). Recently, tremendous progress has been made in targeting PRRs and STING pathways for breast cancer treatment (67–71).

#### 3.3.1 Toll-Like Receptors (TLRs)

PAMPs or DMAPs recognition by TLRs activates an inflammatory response that promotes the elimination of cancer cells in the host (72). There is evidence that TLR signaling can abrogate immunosuppression by reducing myeloid-derived suppressor cells (MDSCs) while upregulating stimulatory molecules, including CD40, CD80, CD86, and pro-inflammatory cytokines production. These signals can further stimulate a more potent nonspecific innate immunity and bridge the adaptive immune responses against breast cancer (73–75).

Among all these TLR-agonists, poly (I: C) was reported to effectively modulate MDSCs activity in a mice model of breast cancer (76). Poly (I: C) directly bond to TLR3 expressed on the surface of MDSCs, therefore, caused the reduction of circulating and infiltrating MDSCs in breast tumors. In addition, TLR7/8 agonist R484 is another successful adjuvant in breast cancer. The use of R484 alone has been demonstrated to retard tumor growth (68). After repeated intraperitoneal injection of R484, the vasculature density of breast tumors was reduced, and tumor cell apoptosis was induced significantly.

Recently, Zheng et al. reported utilizing a vaccine-based nanosystem as an innate immune stimulator for treating breast tumors (77). They confirmed the importance of TLR7 in APC activation, and the nano-sized innate immune stimulator activated the endosomal TLR7 signaling to promote antigen cross-presentation. Furthermore, it can be potentially used as cancer vaccine adjuvants or immunotherapeutic agents to improve immune checkpoint blockade (ICB) immunotherapy.

#### 3.3.2 RIG-I-Like Receptors (RLRs)

RLRs family includes RIG-I and melanoma differentiation-associated gene 5 (MDA5), both of which are cytoplasmic RNA sensors (78). The RNA recognition of RLRs can effectively inhibit breast cancer growth by inducing apoptosis in the IFN-independent manner (79). For example, RIG-I activation mimics viral infection in various cancer types, including breast cancer. The activated RIG-I signaling is associated with NK cell attack, phagocytosis of apoptotic tumor cells, and other immunogenic forms of tumor inhibition (80, 81).

More recently, Domankevich et al. applied an intra-tumoral cytoplasmatic delivery agent to directly activate RLR while bypassing endosomal recognition *via* TLRs (82). They showed the reduced 4T1 triple-negative breast tumors, and the metastasis development was retarded after re-challenge. Another similar study was conducted to deliver poly (I: C) to the cytoplasm in an MDA5-dependent manner while bypassing the TLR3-mediated signaling pathway (69). Furthermore, three human breast cancer cell lines, including MCF-7, MDA-MB-231, and BT-549, were tested to transfect poly(I: C). The transfected cells showed less tumorigenicity when implanting for breast tumor growth. Together, these results suggested that innate adjuvant receptors are emerging targets for antitumor drug therapies.

#### 3.3.3 NOD-Like Receptors (NLRs)

NLRs are cytosolic receptors that detect intracellular pathogens and endogenous byproducts from tissue injury. In recent years, targeting innate immune NLR pathways for cancer immunotherapy, including breast cancer, has attracted increasing attention, due to their multiple functions in intracellular pathogens detection, initiation of inflammation, and the regulation of tissue repair (83). One such example is that NOD1 has been investigated in breast tumor models and showed protective function in breast tumorigenesis (84). Mice inoculated with NOD1-deficient MCF-7 breast cancer cells exhibited rapid growth and continuous malignancy. Another aggressive Hs578T triple-negative breast cancer cell line was also designated to study the impact of NOD1 and NOD2 receptors (70). In this model, NOD1 overexpression reduced estrogen-induced tumor proliferation, while NOD2 activation led to a remarkable suppression of tumor growth. Numerous findings suggested that NLRs and their downstream signaling factors switch roles during different stages of cancer development, including initiation, elimination, and maintenance (83). Thus, it is necessary to elucidate the immune-stimulatory properties of NLRs in breast cancer and potentially modulate treatment outcomes.

#### 3.3.4 C-Type Lectins Receptors (CLRs)

CLRs play a crucial role in facilitating antigen uptake and presentation by MHC molecules (71). Dectin-1 is one typical CLR that can be activated by beta-glucan to regulate the antitumor immune responses. The dectin-1 receptors were expressed mainly on macrophages, neutrophils, dendritic cells, and a subpopulation of T-lymphocytes. Beta-glucan binding to the lectin site of neutrophils or NK cells generated a primed state of the receptor, capable of recruiting tumoricidal granulocytes for cytotoxic killing (71, 85). Oral administration of beta-glucan has been shown to stimulate peripheral blood monocytes expansion and activation in patients with metastatic breast cancer (86), similarly, another clinical trial suggested β-glucans as useful adjuvants in improving life quality for breast carcinoma patients during chemotherapy (87). Lectin-like oxidized-low-density lipoprotein (oxLDL) receptor-1 (LOX-1) was reported to induce TNF-α expression, cellular adhesion and trans-endothelial migration of MDA-MB-231 breast cancer cells (88). Besides, LOX-1 expression on DCs was associated with cross-presentation and activation of cytotoxic CD8+ T cells (89, 90), and meanwhile mediated B cell differentiation and migration (91). Moreover, high levels of LOX-1 expression was found on MDSCs from peripheral blood and tumor of cancer patients but not on MDSCs from healthy donors (92). Therefore, future studies remain to be performed to apply these potential CLRs as anti-breast cancer vaccine adjuvants.

#### 3.3.5 Cytosolic Stimulator of Interferon Genes (STING)-Dependent DNA Sensor

STING is recently discovered as an intracellular DNA recognition receptor that can induce type I interferon production and host innate immune activation. Synthetic STING agonists have been found to regulate tumorigenesis and potently induce antitumor immunity in metastatic breast cancer (93). In addition to its direct inhibitory effect in tumor cells, emerging evidence suggested that STING agonists function as vaccine adjuvants to promote therapeutic response.

Among the STING agonists, 5,6-Dimethylxanthenone-4-acetic Acid (DMXAA) was shown to mediate robust antitumor innate immune responses in MMTV-PyMT tumor bearing mice by vessel destruction and amplified immune cell infiltration (94). However, DMXAA was a murine STING restricted agonist and failed in targeting human breast cancer in phase III clinical trials. Based on these findings, several STING agonists have been generated to target human species while preserving the ability to induce strong type I IFN. Cyclic dinucleotides, such as cyclic diguanylate monophosphate (c-di-GMP), cyclic deadenylate (c-di-AMP), and cyclic guanosine monophosphate–adenosine monophosphate (cGAMP), have been shown to improve vaccination in multiple cancer types, including breast tumor (95–97). Similarly, c-di-GMP could inhibit the spread of metastasis and tumor size in 4T1 metastatic breast tumors. *In situ* delivery of a STING-activating cyclic dinucleotide, ADU-S100/MIW815, against established HER2^+^ breast tumors overcame immune tolerance and further induced tumor regression (98). Moreover, nanoparticle delivery of STING-activating cGAMP was recently demonstrated to inhibit tumors in a genetically engineered mouse model of basal-like triple-negative breast cancer C3 (1)Tag, which was insensitive to checkpoint blockade therapy (67). To further explore the function of STING targeted therapy, increasing preclinical experiments and clinical trials are undergoing investigation in breast cancer and other cancer types (99, 100).

### 3.4 Targeting Innate Immunity in Combination With Other Breast Cancer Therapies

The antitumor response induced by one singular innate immune reagent can be limited, hindering potential therapeutic application. Synergistic activation of innate and efferent arms represents the future therapeutic modality owing to the high potency and specificity for translational medicine. Various approaches have been explored to reduce toxicity and facilitate both innate and adaptive antitumor immune responses. STING-deficient mice showed impaired CD8^+^ T cells, and these mice were more vulnerable to tumor progression and resistance to immunotherapy. Thus, STING targeting therapy had a pivotal function in enhancing immune checkpoint blockade response (ICB) (101). Remarkably, in the ICB-resistant breast cancer model, combination treatment of IFN inhibitors and ICB led to complete tumor eradication and survival (102). This study demonstrated that inhibition of the IFN signaling pathway could restore the immune responses to ICB, which largely expands the use of immunotherapies for highly extensive ICB-combination-therapy-resistant tumors.

Moreover, when combined with ionizing radiation, nano-vaccines delivery of STING activator also reversed the immunosuppressive tumor microenvironment, and this combined approach has been proved as safe and effective radioimmunotherapy for primary and metastatic tumors (103). Interestingly, plasmacytoid dendritic cells (pDC) promote immunosuppression within the tumor microenvironment. However, they are the major type I IFN producers in response to TLR7 and TLR9 activation. Inspired by these features, Wu et al. introduced TLR7 ligand imiquimod (IMQ) and the TLR9 ligand CpG to target pDC, and the combined treatment induced a more potent effect compared to the single-agent (104). Collectively, these emerging strategies **(** **Table 1** **)** suggested that rationally designed innate immunostimulatory adjuvants can be potentially efficient and versatile for developing combination immunotherapy for breast cancer.

**Table 1:** Factors and mechanisms related to anticancer innate immunity and summary of agents in the use of breast cancer immunotherapy.

Innate immune pathway/receptor	Classification	Role in cancer immunity	Innate cells	Agent	Ref
Colony-stimulating factor	M-CSF, G-CSF, GM-CSF, IL-3	regulate the proliferation, maturation, and apoptosis of myeloid cell	DC, TAM, MDSC, neutrophils, NK cell	the anti-CSF1R antibody, CSF1R inhibitor	(53)
Interferon	Type I IFN (IFN-α and IFN-β, *etc.*)	maturation of dendritic cells; recruit NK cell and T helper cells; polarize macrophage phenotypes;	Drug: FLAC with GM-CSF	IFN-α2a, IFN-α2b	(41, 105)
	Type II IFN (IFN-γR1 and IFN-γR2)				
TLRs/Toll-like receptors	Cell surface TLRs (TLR1, TLR2, TLR4, TLR5, TLR6, and TLR10)	mediate both immune surveillance and immune tolerance; activates inflammatory response; cancer vaccine adjuvants or immunotherapeutic agent	DC, TAM, MDSC, neutrophils, NK cell, basophils, eosinophils, mast cell	TLR3 agonist PolyI:C, TLR4 agonist LPS, TLR7/8 imiquimod, TLR9 agonist	(69)
	Intracellular TLRs (TLR3, TLR7, TLR8, TLR9, TLR11, TLR12, and TLR13)			CpG ODNs	(68)
RLRs/RIG-I-like receptors	RIG-I and MDA5	promote transcription factor IRF3 and NF-κB; affect the secretion of inflammatory cytokines; promote phagocytosis of apoptotic tumor cells	DC, TAM, MDSC, neutrophils, NK cell, basophils, eosinophils, mast cell	RIG-I activator MK-4621, Bo-112	(78)
					(80, 81)
					(79)
NLRs/NOD-like receptors	NLRA, NLRB, NLRC, NLRP, NLRX	intracellular pathogens detection; initiation of inflammation; regulation of tissue repair; induce type I IFN secretion	DC, TAM, MDSC, neutrophils, NK cell, mast cell	NOD-2 agonist mifamurtide	(83).
CLRs, C-type lectin receptors	Dectin-1	facilitate antigen uptake and presentation by MHC molecules; recruiting tumoricidal granulocytes	DC, TAM, neutrophils	CMB305, imprime PGG.	(71, 85)
CDS, Cytosolic DNA sensors	STING-dependent Sensors	promote AIM2 inflammasome activation; induce the production of pro-inflammatory cytokines	DC, TAM, MDSC, mast cell	STING agonists MK-1454, MIW815, DMXAA	(94)
					(98)

## 4 Clinical Applications and Current Trials of Innate Immunity-Based Breast Cancer Therapy

So far, several breast cancer immunotherapies have broken immune tolerance in clinical applications, giving novel promises for cancer therapy. We expansively summarized the clinical studies targeting innate immunity for breast cancer therapy (**Table 2**). Several clinical applications targeting innate immunity, using colony-stimulating factors, stimulator of interferon genes along with their agonistic ligands for toll-like receptors, showed these clinical treatments not merely regard as vaccine adjuvants but as well as antitumor agents. Moreover, combination therapies have revealed superior efficacy, may offer a new promise for the development of immune therapy. Herein, we focus on completed or ongoing clinical trials of innate immunity-based breast cancer therapy. Currently there are 16 clinical trials from phase 1 to 4 using colony-stimulating factors (CSF) together with chemotherapy or radiotherapy for breast cancer treatment. CSF was mainly used for enhance the efficacy of chemotherapeutic drugs such as paclitaxel, ciprofloxacin and reduced the neutropenia caused by those drugs. The safety of CSF or PEGylated CSF has been verified in human being. The clinical trials of interferons and toll-like receptors are mainly at stage 1 or 2 to study the safety and optimize the tolerated doses. There is also one stage 1 trial using cytosolic STING-dependent drug to determine the side effects and safety.

**Table 2:** Clinical applications and current trials of innate immunity-based breast cancer therapy.

Target	Phase	Clinical status	Summary	Interventions	Clinical trial identifiers
Colony-stimulating factors	1	Active, not recruiting	Patients’ tumor cells were used to formulate a vaccine to stimulate their immunity by secreting granulocyte-macrophage colony-stimulating factor (GM-CSF).	Biological: Autologous, Lethally Irradiated Breast Cancer Cells	CT00317603
Colony-stimulating factors	2	Completed	GM-CSF was used to improve chemotherapy-induced toxicity in metastatic/locally advanced breast cancer.	Drug: FLAC with GM-CSF	NCT00001239
Colony-stimulating factors	3	Completed	FLAC chemotherapy and GM-CSF or PIXY321 for patients with locally advanced breast cancer.	Drug: FLAC chemotherapy with GM-CSF	NCT00001338
Colony-stimulating factors	1	Completed	Treatment of IL-3 alone or in combination with GM-CSF after FLAC chemotherapy was conducted to study the maximal dose of IL-3 in patients with metastatic breast cancer.	Drug: IL-3	NCT00001269
Colony-stimulating factors	1	Completed	Treatment of paclitaxel (weekly) combined with radiotherapy (biweekly) was performed to study toxicity and response rate.	Drug: Paclitaxel, Vinorelbine, Filgrastim	NCT00724386
				Radiation: Radiation	
Colony-stimulating factors	3	Completed	The biosafety and efficacy of injecting GSF fusion protein and recombinant human serum albumin were studied for preventing chemotherapy-induced neutropenia.	Drug: rHSA-GCSF and G-CSF	NCT03251768
Colony-stimulating factors	4	Completed	Treatment of prophylaxis combined with either ciprofloxacin or G-CSF was conducted to diminish chemotherapy-induced febrile neutropenia.	Drug: Ciprofloxacin	NCT02816112
				Drug: Neupogen	
Colony-stimulating factors	4	Not yet recruiting	Post chemotherapy, patients received a second injection of PEG-rhG-CSF to prevent bone marrow suppression and/or febrile neutropenia in breast cancer.	Drug: PEG-rhG-CSF	NCT04477616
Colony-stimulating factors	1	Completed	Treatment of different doses of rHSA/GCSF to study the safety and tolerance in breast cancer patients.	Drug: rHSA/GCSF	NCT03246009
Colony-stimulating factors	4	Not yet recruiting	Treatment of PEG-rhG-CSF as the primary prevention of neutropenia to study the clinical efficacy, tolerance, and safety in early breast cancer patients.	Drug: PEG-rhG-CSF	NCT04009941
Colony-stimulating factors	2	Completed	To study tumor response rate and survival rate in patients after GM-CSF treatment.	Drug: Herceptin	NCT00429104
				Drug: GM-CSF	
Colony-stimulating factors	2	Completed	To determine the dose and safety of combined rintatolimod and GM-CSF treatment.	Biological: HER-2/neu peptide vaccine	NCT01355393
				Biological: sargramostim, Rintatolimod	
Colony-stimulating factors	1	Completed	To determine the dose and safety of co-treatment of vaccine, Montanide ISA-51 and sargramostim for stage IV breast cancer patients.	Biological: incomplete Freund’s adjuvant, Sargramostim	NCT00079157
				Biological: telomerase: 540-548 peptide vaccine	
Colony-stimulating factors	3	Completed	To study the side effect and efficacy of administering NeuVax™ with sargramostim (GM-CSF).	Biological: NeuVax™ vaccine, Leukine^®^ (sargramostim, GM-CSF)	NCT01479244
Colony-stimulating factors	2	Completed	To determine the efficacy of vaccine and sargramostim (GM-CSF) in post-surgical female patients diagnosed as stage II/III breast cancer with metastasis to the lymph nodes.	Biological: recombinant fowlpox-CEA(6D)/TRICOM vaccine, recombinant vaccinia-CEA(6D)-TRICOM vaccine, Sargramostim	NCT00052351
				Drug: cyclophosphamide, Doxorubicin hydrochloride, Paclitaxel	
				Radiation: radiation therapy	
Colony-stimulating factors	2	Completed	To compare the efficacy of the GP2 peptide vaccine and GM-CSF in breast cancer patients.	Biological: GP2 peptide + GM-CSF vaccine, GM-CSF (sargramostim), AE37 + GM-CSF vaccine,	NCT00524277
Interferons	2	Terminated	To determine the combined efficacy of capecitabine and interferon alfa-2a in breast cancer patients with recurrent or progressive brain metastases.	PEG-interferon alfa-2a	NCT00227656
				Drug: Capecitabine	
Interferons	2	Recruiting	To optimize the dose and safety of delivering interferon-gamma and paclitaxel/trastuzumab/pertuzumab in HER2 positive breast cancer patients.	Biological: Interferon-gamma	NCT03112590
				Drug: Paclitaxel, Trastuzumab, Pertuzumab	
Interferons	1	Recruiting	To explore the safe dose range of IFN-α-2a and the human tolerance index.	Drug: IFN-α-2a	NCT04522557
Interferons	2	Completed	To determine the efficacy of combining cyclophosphamide and tumor cell vaccine in cancer participants at high risk of recurrence.	Biological: allogeneic tumor cell vaccine, autologous tumor cell vaccine, recombinant interferon alfa, recombinant interferon-gamma, sargramostim	NCT00002475
				Drug: cyclophosphamide	
Interferons	1	Completed	To determine the safety and optimal dose of treating interferon alfa in participants with stage IV solid tumors (breast cancer), lymphoma, or myeloma.	Biological: recombinant interferon alpha-1b	NCT00276536
				Drug: IFN	
Toll-like receptors	2	Completed	To study the optimal dose and efficacy of Imiquimod together with cyclophosphamide and radiotherapy in participants with breast cancer and skin metastases.	Radiation: Radiation	NCT01421017
				Drug: Imiquimod	
				Drug: Cyclophosphamide	
Toll-like receptors	1	Terminated	To study the dose range of cyclophosphamide, pegfilgrastim, and TLR8 agonist VTX-2337 (CyNeuMoto).	Drug: Cyclophosphamide	NCT02650635
				Biological: Pegfilgrastim, TLR8 Agonist VTX-2337	
Toll-like receptors	2	Completed	To study the side effect and efficacy of Imiquimod in patients with breast cancer and metastases to skin or chest wall recurrences.	Drug: Imiquimod	NCT00899574
Toll-like receptors	2	Completed	To determine the safe dose of Imiquimod together with Abraxane.	Drug: Imiquimod, Abraxane	NCT00821964
Toll-like receptors	2	Completed	To study the efficacy of 852A in metastatic breast cancer patients.	Drug: 852A	NCT00319748
Toll-like receptors	1	Terminated	To study the immunogenicity of administering a TLR3 vaccine together with poly-ICLC in patients with stage I.B. to IIIA breast cancer.	Biological: poly-ICLC	NCT01532960
				Biological: Peptides from Her-2/neu, CEA, & CTA, Peptide-te	
Cytosolic STING-dependent	1	Recruiting	To determine the side effect and efficacy of E7766 by intratumorally administrated in patients with advanced solid tumors or lymphomas.	Drug: E7766	NCT04144140

## 5 Conclusion and Future Directions

The innate immunity exerts essential roles in shaping breast cancer development and altering tumor microenvironment through the recruitment of circulating innate immune cells and trigger the activation of adaptive immune cells, or through the functional skewing of residential immune cells by the secreted cytokines, growth factors and PRRs-mediated interaction. To understand the breast tumor microenvironment, preclinical studies have been shown that innate immune cells and the secreted cytokines have multifaceted roles that either promote or suppress breast tumor during disease progression or therapeutic responses. For instance, when upon interaction with tumor antigen *via* TLR or STING that primarily expressed on DCs and macrophages, these stimulated innate immune cells undergo maturation or repolarization (e.g. immature DCs differentiate into mature DCs; pro-tumoral TAMs repolarize towards anti-tumoral M1 macrophages). They secrete IL-12 and type I IFNs that can prime and activate tumor specific CD8^+^ T cells for cytotoxic killing against breast tumor cells. The secreted IL-12 and type I IFNs also can activate NK cells for lysing tumor cells. Meanwhile, breast tumor cells synthesize and secret TGFβ that directly or indirectly target various immune cells, which altogether mediate the immune escape for invasion and metastasis (46). This also leads to another opportunity of developing routine immunoprofiling analysis of breast tumors and patients, which should be matched to effective strategy during treatment planning and prognosis. Therefore, understanding the interaction among innate immune cells with breast tumor microenvironment can aid to select therapeutic strategy for achieving successful outcomes based on the personalized immunoprofiling information.

Although T cell-based immunotherapies have achieved breakthrough outcomes in many cancer types, including some breast cancers, patients with poor infiltrating lymphocytes had limited or no response. To tackle the difficult-to-treat breast cancers or enhance the antitumor responsiveness of immunotherapy, numerous preclinical studies and early clinical trials have been conducted to activate innate immune cell populations or sensing pathways alone or in combination with adaptive immune therapies. One clinical study NCT02981303 is conducted on metastatic triple negative breast cancer (TNBC) patients to test the combination of Dectin Receptor agonist Imprime PGG and the immune checkpoint blocker pembrolizumab. They showed robust infiltration of activated myeloid cells and TILs associated with promising synergistic anti-tumor effects. Another ongoing phase I trial NCT03841110 is conducted to evaluate the combined effect of adoptive transferred NK cells (iPSC-derived) and anti-PD-1 antibodies (nivolumab or pembrolizumab). Although the trial NCT03172936 was recently terminated, their preliminary results suggest that STING agonist ADU-S100 in combination with the PD-1 blocker spartalizumab was well tolerated and mediated some clinical activity in patients with PD-1-naïve TNBC (99, 106). Therefore, increasing progress is being made to manage patient safety and determining nontoxic doses of single-agent or combination drugs that exhibit effective antitumor responses. Current data encourages a continued exploration of these strategies for translational application.

## Data Availability Statement

The original contributions presented in the study are included in the article/supplementary material. Further inquiries can be directed to the corresponding authors.

## Author Contributions

NC designed this study. YY, CX, FC, and QL wrote the manuscript. NC revised the manuscript. All authors contributed to the article and approved the submitted version.

## Funding

The authors’ work is supported by a grant from the United States National Cancer Institute/National Institute of Health (NCI/NIH K00 CA223019 to NC).

## Conflict of Interest

Author YY is/was employed by Zenomics. Inc.

The remaining authors declare that the research was conducted in the absence of any commercial or financial relationships that could be construed as a potential conflict of interest.

## Publisher’s Note

All claims expressed in this article are solely those of the authors and do not necessarily represent those of their affiliated organizations, or those of the publisher, the editors and the reviewers. Any product that may be evaluated in this article, or claim that may be made by its manufacturer, is not guaranteed or endorsed by the publisher.
