# Corrigendum: Targeting innate immunity in breast cancer therapy: a narrative review

**DOI:** 10.3389/fimmu.2023.1353708

**Published:** 2023-12-20

**Authors:** Yanqi Ye, Chun Xu, Fengqian Chen, Qi Liu, Ning Cheng

**Affiliations:** ^1^ Zenomics. Inc. Magnify at California NanoSystems Institute, Los Angeles, CA, United States; ^2^ School of Dentistry, The University of Queensland, Brisbane, QLD, Australia; ^3^ School of Medicine, University of Maryland, Baltimore, MD, United States; ^4^ School of Medicine, Johns Hopkins University, Baltimore, MD, United States; ^5^ Department of Otolaryngology - Head and Neck Surgery, University of California at San Francisco, San Francisco, CA, United States

**Keywords:** innate immunity, breast cancer, cancer immunotherapy, clinical studies, vaccine adjuvants

In the published article, there was a missing citation of “The Crosstalk Between Tumor Cells and the Immune Microenvironment in Breast Cancer: Implications for Immunotherapy, doi.org/10.3389/fonc.2021.610303”in the legend for **Figure 1** as published. The corrected **Figure 1** legend appears below.

**Figure 1:**
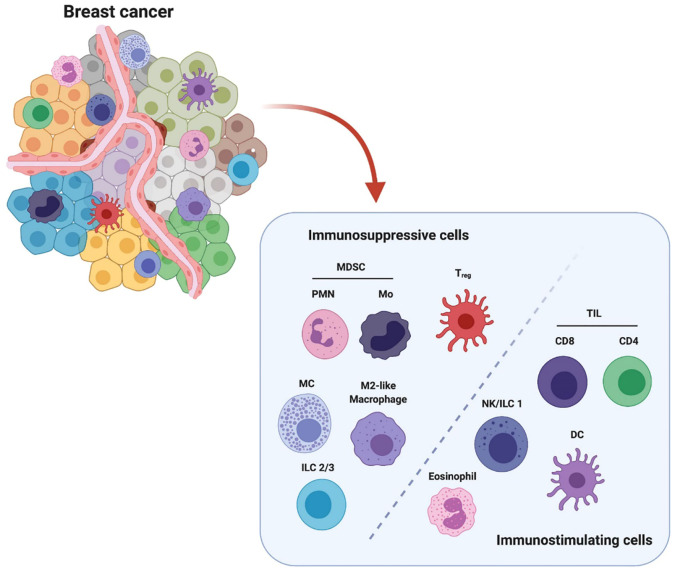
Breast cancer microenvironment is populated by diverse infiltrating immune cells. These immune cells are categorized into immunosuppressive population and immunostimulating population (e.g.) according to their major characteristics in modulating breast cancer. The immunosuppressive cells include polymorphonuclear myeloid-derived suppressor cells (PMN-MDSCs), monocytic MDSC (Mo-MDSC), regulatory T cells (Treg), mast cells (MC), M2-like macrophages and type 2/3 innate lymphoid cells (ILC 2/3). The immunostimulating cells include tumor infiltrating lymphocytes CD8+ and CD4+ T cells, nature killer cells/type 1 innate lymphoid cells (NK/ILC1), dendritic cells (DCs) and eosinophils. This figure is reproduced with permission from “The Crosstalk Between Tumor Cells and the Immune Microenvironment in Breast Cancer: Implications for Immunotherapy, doi.org/10.3389/fonc.2021.610303” Copyright **^©^2021 Frontiers**.

The authors apologize for this error and state that this does not change the scientific conclusions of the article in any way. The original article has been updated.

